# Short-term functional and oncological outcomes of intralesional curettage supplemented by bone cementation for chondroblastoma : a cross-sectional study

**DOI:** 10.1097/MS9.0000000000003191

**Published:** 2025-03-28

**Authors:** Hassan Mohammed Hassan Elbahri, Hozifa Mohammed Ali AbdElmaged, Musa Yassin Mohamed Awad, Yousif Omer Elgaili Yousif, Sara Gamareldein A. Khalafalla

**Affiliations:** a Department of Surgery, College of Medicine and Health Sciences, Arabian Gulf University (AGU), Bahrain; b Faculty of Medicine, International University of Africa (IUA), Khartoum, Sudan; c Assistant Professor of Orthopaedics, Alzaiem Alazhari University, Alhalfaya, Khartoum north, Khartoum, Sudan. Tel.: +249913069716.; d Senior Orthopaedics Resident, Sudan Medical Specialization Board, Khartoum, Sudan; e Surgery Department, Alzaiem Alazhari University, Khartoum, Sudan; f Paediatric Specialist, Ministry of Health, Khartoum, Sudan

**Keywords:** bone cementation, chondroblastoma, intralesional curettage, oncology

## Abstract

**Background:**

Chondroblastoma is a rare, benign neoplasm accounting for less than 1% of all primary bone tumors. It is treated with complete surgical curettage with or without chemical or physical adjuvants. The present study aims to assess the functional and oncological outcomes of patients with chondroblastoma treated with intralesional curettage supplemented by bone cementation of the resulting bone defect.

**Materials and methods:**

This study is an observational, descriptive, prospective hospital-based study conducted from April 2022 to August 2022 at the Ibrahim Malik Teaching Hospital and Future Hospital in Khartoum State, Sudan. It included patients with chondroblastoma who were treated with intralesional curettage and subsequent filling of the remaining bone cavity with bone cement. The researchers collected data from the patients’ hospital records, as well as with the aid of the Musculoskeletal Tumor Society Scoring System, and analyzed them using SPSS V 28.

**Results:**

The study population comprised 32 patients with a mean age of 20 ± 6 years; the majority were males, 62.5% (*n* = 20), and students by occupation, 66% (*n* = 21). The proximal tibia was the most commonly involved site, corresponding to 59% of the cases (*n* = 19). Functional evaluation using the Musculoskeletal Tumor Society Scoring System revealed a statistically significant improvement from a preoperative score of 62 ± 10 to a postoperative score of 91 ± 3 (*P* value = 0.001). No recurrence or need for amputation was reported. Almost half of the study participants, 47% (*n* = 15) experienced moderate to severe pain. Postoperatively, 97% (*n* = 31) were satisfied with pain relief, and all patients were satisfied with the procedure. Functional outcomes improve with time after surgery.

**Conclusion:**

The study showed a favorable outcome regarding pain relief and functional restoration in patients with chondroblastoma. The oncological results were also satisfactory; no recurrence, or need for amputation was recorded.

## Introduction

**Highlights**

- This cross-sectional study of 32 Sudanese patients with chondroblastoma demonstrates favorable short - term (< 6m) functional and oncological outcomes following intralesional curettage supplemented by bone cementation of the remaining bone cavity.
- Despite initial moderate to severe postoperative pain in most patients, significant functional improvement was observed, with a mean Musculoskeletal Tumor Society Score increasing from 62 preoperatively to 91 postoperatively (*p*< 0.001). Notably, no recurrences or amputations were reported, and patient satisfaction regarding pain relief and the procedure was high.
- These findings underscore the effectiveness of intralesional curettage and subsequent bone cementation of the resulting bone defect in achieving both local tumor control and favorable functional outcomes for patients with chondroblastoma.

The benign neoplasm, chondroblastoma, which generates chondroids, consists of chondroblasts. It accounts for less than 1% of all bone malignancies and generally originates in the apophyses, or epiphyses of skeletally immature patients^[1]^. Additionally, it makes up 9% of all benign bone tumors^[2-4]^. Pain is the most frequent and potentially persistent presentation. Other signs and symptoms include limping, localized edema, effusion, and restricted joint mobility^[1,5,6]^. Chondroblastoma is often detected by imaging in the epiphyses of long bones. Furthermore, it might have metaphyseal extension, most likely by extension of the physis^[7-9]^. It often manifests as a small- to medium-sized lesion that is confined, eccentric, and lytic with a thin rim of sclerotic bone, measuring 3–6 cm on average^[10,11]^. The histological features of chondroblastoma include the proliferation of chondroblasts and other components, including mature cartilage, sporadic multinucleated giant cells, calcification, and occasionally areas of aneurysmal bone cyst^[12]^.

The primary form of treatment for chondroblastoma is surgery, which involves sufficient intralesional curettage either by itself or in conjunction with chemical (phenol) or physical (cryosurgery or cementation) adjuvants^[13]^. The resulting defect is often filled with bone graft or cement. En-bloc resection is an option in some circumstances; however, it is not always necessary. Rates of local recurrence range from 10% to 30%. Although rare, examples of benign pulmonary metastases and malignant transformation of recurring tumors have also been reported^[14-16]^. The level of bone and or joint involvement, the lesion’s anatomic position, and the staging, all affect surgical care. Stage 1 (latent) or stage 2 (active) may indicate intralesional excision, whereas stage 3 (aggressive) is a reason for marginal, or broad resection^[17,18]^. Chemical cauterizations with phenol or cryosurgery are examples of adjunctive therapy. Following surgical curettage, bone grafting, and cryotherapy reduce the probability of recurrence. Adjuvant radiation and chemotherapy have no established roles. Resection remains the preferred course of action for new tumors^[1]^.

Chondroblastoma is characterized by aggressive growth, possible recurrence after surgical treatment, and, in rare cases, metastases. Surgical management is the primary treatment, including intralesional curettage with or without adjuvants. It is believed that the intraoperative use of adjuvants destroys all tumor cells; it can also be used as a predictor to monitor tumor recurrence on radiographs^[19]^.

The prognosis for chondroblastoma is generally good, and surgery is frequently followed by complete recovery^[20,21]^.

To date, several studies have evaluated the effectiveness of intralesional curettage supplemented by bone cementation of the resulting bone defect in the treatment of chondroblastoma, but no local studies support or reject this treatment modality in Sudanese patients. Therefore, the present study aims to assess the functional and oncological outcomes of patients with chondroblastoma treated with intralesional curettage and subsequent bone cementation of the remaining bone cavity in Khartoum state.

## Materials and methods

This study is an observational descriptive cross-sectional hospital-based study. It was carried out from April 2022 to August 2022 at the Ibrahim Malik Teaching Hospital and Future Hospital in Khartoum State, Sudan. The methodology in this study has been reported according to the STROCSS 2021 Guidelines^[22]^.

All patients with chondroblastoma who presented to the orthopedic oncology unit of the aforementioned hospital during the study period and were submitted to intralesional curettage of the tumor supplemented by bone cementation of the resulting bone defect were included in the study population. Patients who received other treatment modalities, such as bone grafting, curettage alone, or showed any histological signs of a malignant transformation, were excluded. Data were collected using a structured questionnaire by the researcher, specifically the Musculoskeletal Tumor Society Scoring System (MTSS). The patients were evaluated at the time of presentation, preoperatively, intraoperatively, and postoperatively. The MTSS was developed in 1993 as an objective tool to measure functional outcomes in patients affected by neoplasms. Even if the MSTS score has never been adequately validated in its original version, it is widely used in clinical practice^[23]^. In the study presented herein, the researcher evaluated the functional outcome in chondroblastoma patients treated with intralesional curettage of the tumor and subsequent bone cementation of the remaining bone cavity with the aid of MTSS after a mean postoperative follow-up period of 3 months (3 ± 2 months).

Data were entered into a Microsoft Excel data sheet and analyzed using SPSS version 28 software. Categorical variables were represented in the form of frequencies and proportions. Continuous variables were expressed as mean and standard deviation in each case. Paired *T*-test and multivariate linear regression test were used to assess significance. The *P* value (probability that the result is true) of <0.05 was considered statistically significant after assuming all the rules of the statistical tests and the confidence level. Data presentation after analysis was performed by means of univariable tables, cross-tabulation (bivariable tables), figures, and narrative illustrations.

Written ethical clearance and approval for conducting this research were obtained from the Sudan Medical Specialization Board Ethical Committee (EDC) under the ID SMSBA1154221. Informed consent was provided by the patients’ parents and/or legal guardians for those under 18 years. The patient data collected in terms of this study was used only for research purposes. Privacy issues were intentionally considered. Participation was voluntary. Any participant had the right to withdraw at any stage.

## Results

This study included 32 Sudanese patients treated for chondroblastoma with intralesional curettage followed by bone cementation of the remaining bone cavity. The patients’ mean age was 20 ± 6 years; the majority were male, 62.5% (*n* = 20), and students by occupation, 66% (*n* = 21), as shown in Table 1.

**Table 1:** Demographic data of patients treated with intralesional curettage and bone cement in the Khartoum State (*n* = 32)

Demographics	Frequency	Percent
Age		
0–10	1	3%
11–20	16	50%
21–30	14	44%
31–40	1	3%
Gender		
Male	20	62.5%
Female	12	37.5%
Occupation		
Employee	2	6%
Free worker	7	22%
Housewife	2	6%
Student	21	66%

In 44% of cases, there was a history of preceding trauma (*n* = 14), pain was the main presentation in 91% (*n* = 29), and one-third presented with swelling in 34% (*n* = 11). The proximal tibia (*n* = 19) was the most commonly involved site (59%). A histopathological diagnosis of chondroblastoma was made in 84% of patients. The patients were followed up over a period of 3 ± 2 months postoperatively, as shown in Table 2.

**Table 2:** Clinical findings of chondroblastoma patients treated with intralesional curettage and bone cement in Khartoum State (*n* = 32)

Clinical findings	Frequency	Percent
History of trauma	14	44%
Presentation		
Pain	29	91%
Swelling	11	34%
Site		
Distal femur	5	16%
Proximal femur	6	19%
Proximal tibia	19	59%
Talus	2	6%
Side		
Right	19	59%
Left	13	41%
Histopathological diagnosis	27	84%
Metastasis	0	0%

Preoperatively, antibiotics were administered in 84% (*n* = 27). No intraoperative complications were reported. Hospital stay was 2 ± 0.5 days. No surgical wound infection was reported, and motion was started in 4 ± 1 days, as shown in Table 3. More specifically, both passive and active range of motion exercises started gradually after 2 weeks of suture removal, focusing on joint mobility and rehabilitation. Weight-bearing activities were introduced gradually as part of the rehabilitation process to ensure optimal recovery and functional outcomes.

**Table 3:** Preoperative, intraoperative, and postoperative events of chondroblastoma patients treated with intralesional curettage and bone cement in Khartoum State (*n* = 32)

	Frequency	Percent
Preoperative		
Antibiotics	27	84%
Tranexamic acid	0	0
Intraoperative		
Blood loss	0	0
Nerve energy	0	0
Transfusion	0	0
Reaction	0	0
Postoperative		
Hospital stay (days)	2 ± 0.5	
Wound infection	0	0
Started ROM (days)	4 ± 1	

ROM, range of motion.

Functional evaluation using the MTSS revealed improvement from a preoperative score of 62 ± 10 to a postoperative score of 91 ± 3, which was statistically significant (*P* value = 0.001), as shown in Table 4.

**Table 4:** Musculoskeletal Tumor Society Scoring System score of chondroblastoma patients treated with intralesional curettage and bone cement in Khartoum State (*n* = 32)

	Preoperative	Postoperative	Difference	Significance
Pain	1.38 ± 1	4.94 ± 0.2	3.5	0.001
Function	2.28 ± 1.5	4.66 ± 0.7	2.3	0.001
Emotional	3.78 ± 0.6	2.94 ± 0.3	−0.8	0.001
Support	4.5 ± 1	5	0.5	0.01
Walking	3.22 ± 0.4	4.94 ± 0.7	2.7	0.001
Gait	3.44 ± 0.7	4.81 ± 0.5	1.3	0.001
Total	18.6 ± 3	27.3 ± 1	9	0.001
Total percent	62 ± 10	91 ± 3	28.8	0.001

**Figure 1.**
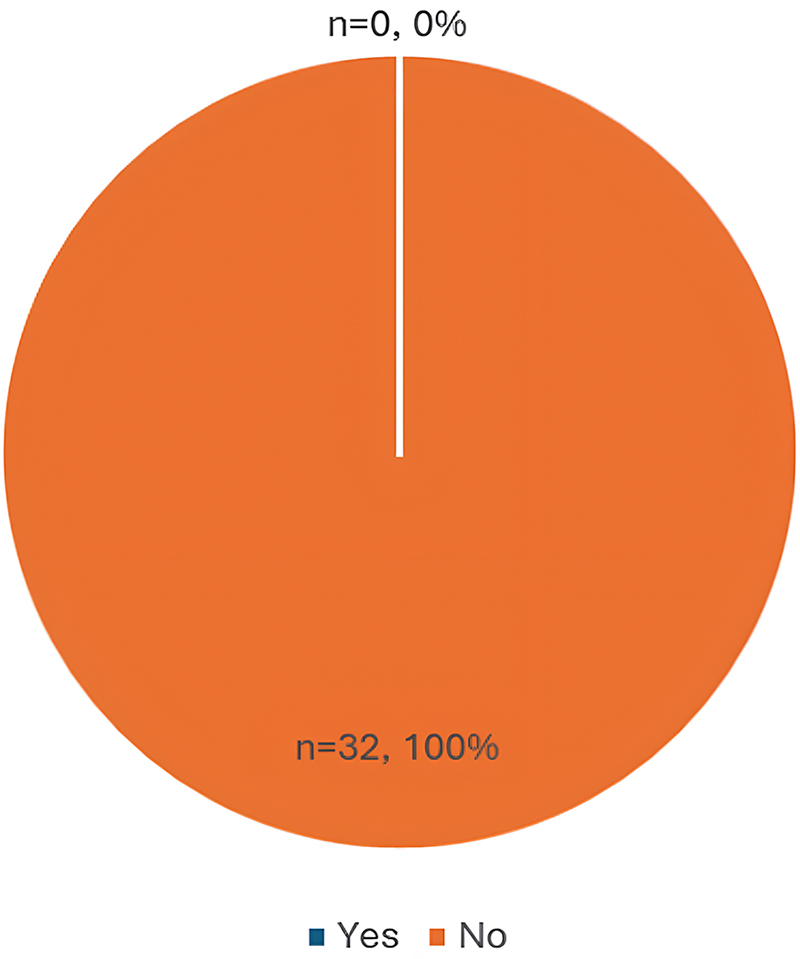
Recurrence among patients with chondroblastoma treated with intralesional curettage and bone cement in the Khartoum state (*n* = 32).

**Figure 2.**
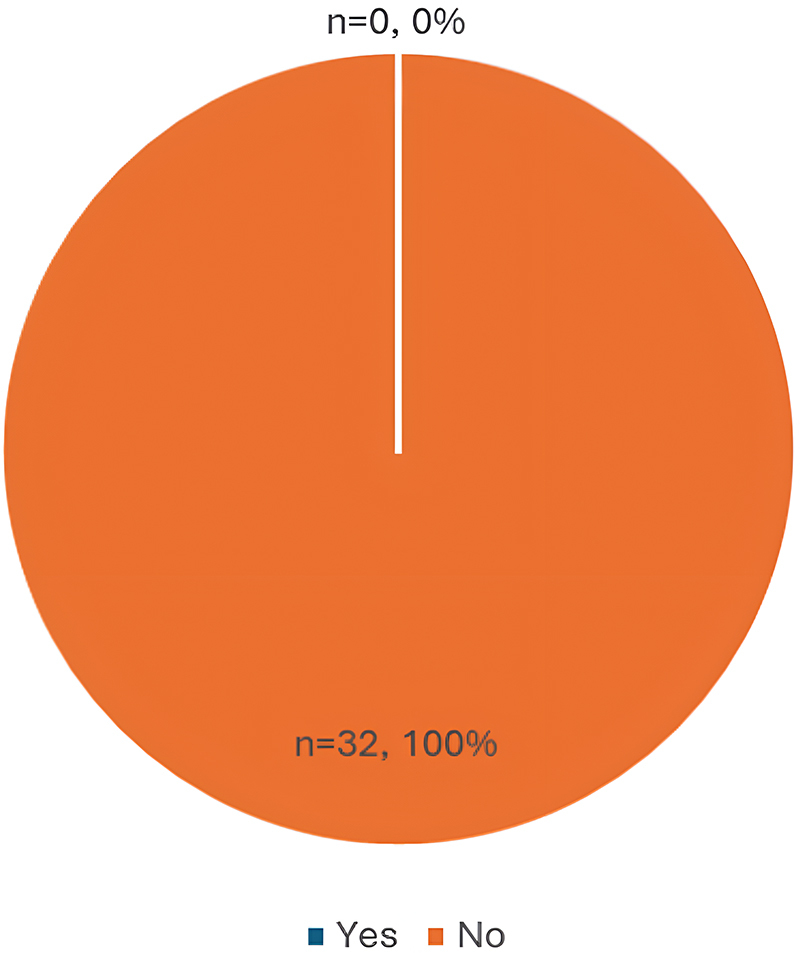
Need for amputation among chondroblastoma patients treated with intralesional curettage and bone cement in Khartoum state (*n* = 32).

Regarding the oncological outcomes, no recurrence or need for amputation was reported, as shown in Figs. 1 and 2.

**Figure 3.**
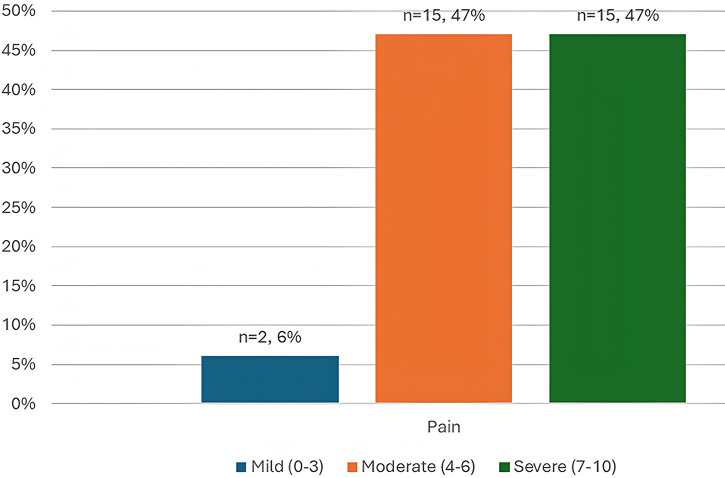
Pain assessment among patients with chondroblastoma treated with intralesional curettage and bone cement in the Khartoum state (*n* = 32).

**Figure 4.**
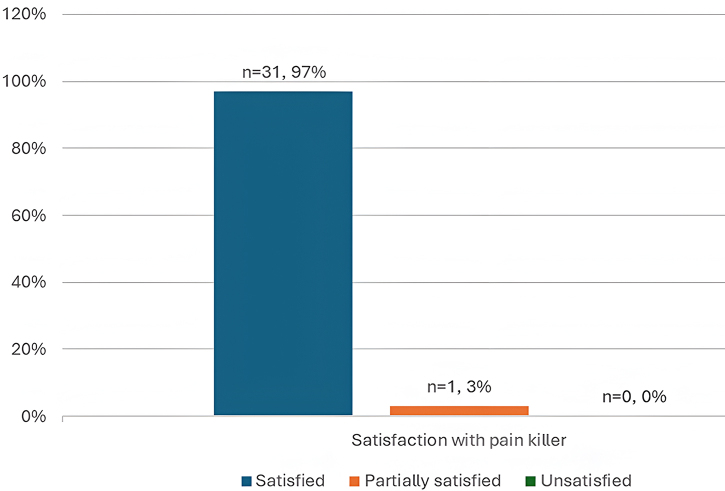
Satisfaction with pain relief among chondroblastoma patients treated with intralesional curettage and bone cement in Khartoum state (*n* = 32).

**Figure 5.**
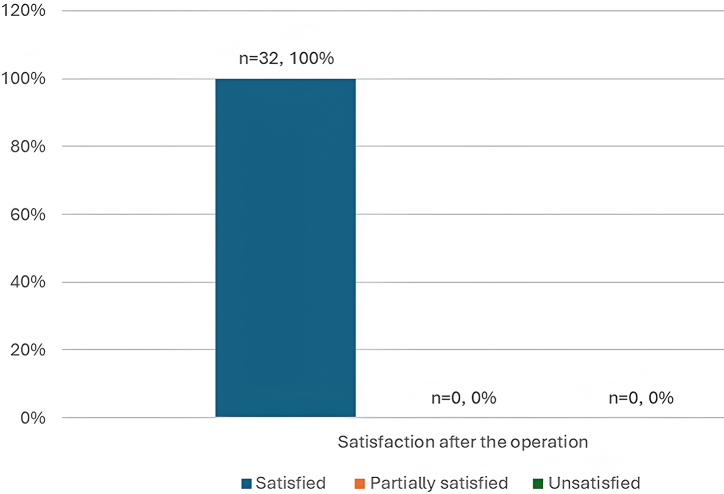
Satisfaction with the operation among patients with chondroblastoma treated with intralesional curettage and bone cement in the Khartoum state (*n* = 32).

As shown in Fig. 3, almost half of the study participants, 47% (*n* = 15) experienced moderate to severe bone pain. Postoperatively, the majority, 97% (*n* = 31), were satisfied with pain relief as shown in Fig. 4. In addition, all patients were satisfied with the procedure, as shown in Fig. 5.

The multivariate linear regression test revealed that functional outcomes improve with time after surgery (*P* value = 0.03), and there was no other significant association, as shown in Table 5.

**Table 5:** Factors affecting functional outcomes of intralesional curettage supplemented by bone cementation (*n* = 32)

	Beta-coefficient	*P* value
Age	−0.3	0.4
Gender	6.6	0.1
History of trauma	1.7	0.7
Time since surgery	1.8	0.03
Preoperative antibiotics	0.6	0.9

## Discussion

This study included 32 Sudanese patients treated for chondroblastoma with intralesional curettage and subsequent bone cementation of the resulting bone defect as a physical adjuvant. Their mean age was 20 ± 6 years, and most of them were males (male-to-female ratio 1.7; 1). Chen *et al.* revealed that most chondroblastomas are diagnosed in the second to third decade of life (mean age, 19–23 years) with a male predominance of 2:1^[1]^. Also, many recent studies consistently reported male predominance and presentation in the second decade of life^[14,18,24-27]^.

In this study, pain was the main presenting symptom. Recent studies mentioned that the presentation of chondroblastoma is bone pain of gradual onset^[1,19,20]^.

The sites predominantly involved in our patients were the proximal tibia, followed by the proximal and distal femur, and there were only two cases of foot involvement. Ebeid *et al.* have also reported that the proximal tibia, the proximal femur, and the humerus were the sites most commonly affected by chondroblastoma^[24]^. Lehner *et al.* in another study found that the sites involved were the knee joint, the distal femur, and the proximal tibia^[25]^.

No metastasis was found, and no recurrence, need for amputation, or wound infection was reported.

Almost half of the study participants experienced moderate to severe pain. Postoperatively, the vast majority of them (97%) were satisfied with pain alleviation. Similarly to our study, recent studies found that chondroblastoma has a good prognosis, and patients often experience full resolution after surgical treatment^[19,20]^. Ebeid *et al.* reported only a few cases of recurrence^[24]^, and Lehner *et al*. (2011) reported that only one patient experienced local recurrence after primary tumor resection^[25]^. In contrast to these findings, Deventer *et al*. reported a high recurrence rate of 39.5%^[27]^. However, only one patient had the rare malignant metastatic chondroblastoma and eventually died, as reported by Suneja *et al*.^[14]^.

Functional outcomes were evaluated using the MTSS, which revealed an improvement of almost 30% from a preoperative score of 18.6 ± 3 (62 ± 10%) to a postoperative score of 27.3 ± 1 (91 ± 3%). Similarly to our postoperative function score, a recent study by Özer *et al*. reported a mean MTSS score of 27.3 after studying the functional and oncological outcome after chondroblastoma treatment with extended (aggressive) curettage^[18]^. Also similar to our findings, Ebeid *et al*. reported a mean MTSS score of 28.8 after treatment of chondroblastoma^[24]^. Furthermore, Suneja *et al*. found a mean MTSS score of 94.2%^[14]^. Moreover, in the latter study, the analysis of factors affecting functional outcomes revealed that it improves over time.

Undoubtedly, the study presented herein has certain important limitations, including the short follow-up period, the two centers design of the trial, and the fact that 16% of study participants had no histological diagnosis before the definitive treatment as it was their choice after the counseling, they prefer to have the histopathological specimen with the definitive treatment. In addition, the results of this single-center study require confirmation through the conduction of prospective, multicenter studies and, ideally, randomized, controlled trials (RCTs) comparing different forms of chondroblastoma treatment in Sudanese patients. However, this is not feasible at present due to the lack of financing.

## Conclusion

The present study underlined the favorable functional results of intralesional curettage with bone cementation of the remaining bone cavity in patients affected by chondroblastoma, as measured by the MTSS score. The surgical procedure was safe since it had positive postoperative outcomes without complications, recurrence, or malignant transformation. In fact, over time, functional results significantly improved after surgery, confirming the effectiveness of the surgical procedure. All in all, intralesional curettage supplemented by bone cementation seems to be a safe and effective technique for chondroblastoma treatment, with satisfactory oncological results. Future investigations, ideally in terms of properly designed RCTs, should delve into factors impacting functional restoration and procedural complications to enhance patient care and surgical outcomes.

## Data Availability

Available upon reasonable request.

## References

[1] Chen W DiFrancesco LM. Chondroblastoma: an update. Arch Pathol Lab Med 2017;141:867–71. 28557595 10.5858/arpa.2016-0281-RS

[2] Atalar H Basarir K Yildiz Y. Management of chondroblastoma: retrospective review of 28 patients. J Orthop Sci 2007;12:334–40. 17657552 10.1007/s00776-007-1141-2

[3] Strong DP Grimer RJ Carter SR. Chondroblastoma of the femoral head: management and outcome. Int Orthop 2010;34:413–17. 19387641 10.1007/s00264-009-0779-0 PMC2899288

[4] Masui F Ushigome S Kamitani K. Chondroblastoma: a study of 11 cases. Eur J Surg Oncol 2002;28:869–74. 12477480 10.1053/ejso.2002.1276

[5] Turcotte RE Kurt AM Sim FH. Chondroblastoma. Hum Pathol 1993;24:944–49. 8253461 10.1016/0046-8177(93)90107-r

[6] Sailhan F Chotel F Parot R. Sofop. Chondroblastoma of bone in a pediatric population. J Bone Joint Surg 2009;91:2159–68. 19723993 10.2106/JBJS.H.00657

[7] Brien EW Mirra JM Ippolito V. Chondroblastoma arising from a nonepiphyseal site. Skeletal Radiol 1995;24:220–22. 7610417 10.1007/BF00228930

[8] Xu H Ding Y Niu X. Chondroblastoma of bone in the extremities: a multicenter retrospective study. J Bone Joint Surg 2015;97:925–31. 26041854 10.2106/JBJS.N.00992

[9] Maheshwari AV Jelinek JS Song AJ. Metaphyseal and diaphyseal chondroblastomas. Skeletal Radiol 2011;40:1563–73. 21773875 10.1007/s00256-011-1227-y

[10] Chandu Silva M Reid R. Chondroblastoma: varied histologic appearance, potential diagnostic pitfalls, and clinicopathologic features associated with local recurrence. Ann Diagn Pathol 2003;7:205–13. 12913842 10.1016/s1092-9134(03)00048-0

[11] Bloem JL Mulder JD. Chondroblastoma: a clinical and radiological study of 104 cases. Skeletal Radiol 1985;14:1–9. 4023729 10.1007/BF00361187

[12] Kurt AM Unni KK Sim FH. Chondroblastoma of bone. Hum Pathol 1989;20:965–76. 2793161 10.1016/0046-8177(89)90268-2

[13] Lin PP Thenappan A Deavers MT. Treatment and prognosis of chondroblastoma. Clin Orthop Relat Res 2005;438:103–09. 16131877 10.1097/01.blo.0000179591.72844.c3

[14] Suneja R Grimer RJ Belthur MV. Chondroblastoma of bone: long-term results and functional outcome after intralesional curettage. J Bone Joint Surg 2005;87:974–78. 15972914 10.1302/0301-620X.87B7.16009

[15] Ostrowski ML Johnson ME Truong LD. Malignant chondroblastoma presenting as a recurrent pelvic tumor with DNA aneuploidy and p53 mutation as supportive evidence of malignancy. Skeletal Radiol 1999;28:644–50. 10591928 10.1007/s002560050567

[16] Tamura M Oda M Matsumoto I. Chondroblastoma with pulmonary metastasis in a patient presenting with spontaneous bilateral pneumothorax: report of a case. Surg Today 2011;41:1439–41. 21922374 10.1007/s00595-010-4469-8

[17] Angelini A Arguedas F Varela A. Chondroblastoma of the foot: 40 cases from a single institution. J Foot Ankle Surg 2018;57:1105–09. 30368424 10.1053/j.jfas.2018.05.005

[18] Özer D Arikan Y Gur V. Chondroblastoma: an evaluation of the recurrences and functional outcomes following treatment. Acta Orthop Traumatol Turc 2018;52:415–18. 30249436 10.1016/j.aott.2018.07.004 PMC6318575

[19] van der Heijden L Dijkstra PDS van de Sande MAJ. The clinical approach toward giant cell tumor of bone. Oncologist 2014;19:550–61. 24718514 10.1634/theoncologist.2013-0432 PMC4012970

[20] Huvos AG Huvos AG Marcove RC. Chondroblastoma of bone. A clinicopathologic and electron microscopic study. Cancer 1972;29:760–71. 5060653 10.1002/1097-0142(197203)29:3<760::aid-cncr2820290332>3.0.co;2-u

[21] Dahlin DC Ivins JC. Benign chondroblastoma. A study of 125 cases. Cancer 1972;30:401–13. 5051664 10.1002/1097-0142(197208)30:2<401::aid-cncr2820300216>3.0.co;2-b

[22] Mathew G Agha R Albrecht J. STROCSS 2021: strengthening the reporting of cohort, cross-sectional and case-control studies in surgery. Int J Surg Open 2021;37:100430. 10.1016/j.ijsu.2021.106165 34774726

[23] Enneking WF Dunham WK Gebhardt MC. A system for the functional evaluation of reconstructive procedures after surgical treatment of tumors of the musculoskeletal system. Clin Orthop Relat Res 1993;286:241–46. 8425352

[24] Ebeid WA Hasan BZ Badr IT. Functional and oncological outcome after treatment of chondroblastoma with intralesional curettage. J Pediatr Orthop 2019;39:312–17. 10.1097/BPO.0000000000001293 30839485

[25] Lehner B Witte D Weiss S. Clinical and radiological long-term results after operative treatment of chondroblastoma. Arch Orthop Trauma Surg 2011;131:45–52. 20364261 10.1007/s00402-010-1099-y

[26] Penna V Toller EA Ferreira AJ. Results from clinical and radiological follow-up, after surgical treatment of chondroblastoma. Rev Bras Ortop 2011;46:561–64. 27027054 10.1016/S2255-4971(15)30412-2 PMC4799312

[27] Deventer N Deventer N Gosheger G. Chondroblastoma: is intralesional curettage with the use of adjuvants a sufficient way of therapy? J Bone Oncol 2021;26:1–7. 10.1016/j.jbo.2020.100342 PMC7750402 33364155

